# Proteinase-activated receptor-2 mediated inhibition of TNFα-stimulated JNK activation — A novel paradigm for G_q/11_ linked GPCRs

**DOI:** 10.1016/j.cellsig.2009.09.028

**Published:** 2010-02

**Authors:** Kathryn McIntosh, Margaret R. Cunningham, Laurence Cadalbert, John Lockhart, Gary Boyd, W.R. Ferrell, Robin Plevin

**Affiliations:** a Department of Physiology and Pharmacology, University of Strathclyde, Strathclyde Institute for Pharmacy and Biomedical Sciences, 27 Taylor Street, Glasgow, G4 0NR, Scotland, UK; b School of Engineering and Science, University of The West of Scotland, Paisley PA1 2BE, UK; c Centre for Rheumatic Disease, Royal Infirmary, Glasgow G31 2ER, UK

**Keywords:** PAR-2, proteinase-activated receptor-2, PAR-2 AP, PAR-2 activating peptide, 2f-LIGKV-OH, 2-furoyl-LIGKV-hydroxyl, JNK, c-jun N-terminal protein kinase, NFκB, nuclear factor kappa B, PKC, protein kinase C, MAP kinase, mitogen-activated protein kinase, PMA, phorbol 12-myristate 13-acetate, TNFα, tumour necrosis factor-alpha, TNFR1, TNF-receptor-1, GPCR, G-protein-coupled receptors, RIP, receptor interacting protein, TRADD, TNF receptor activated death domain, TRAF, TNF receptor activating factor, NHEK, normal human epithelial keratinocytes, GF109203X, 3-[1-[3-(dimethylamino)propyl]-1H-indol-3-yl]-4-(1H-indol-3-yl)-1H-pyrrole-2,5-dione monohydrochloride, FADD, FAS-associated death domain, MADD, MAP kinase activating death domain protein, IL-6, interleukin-6, Proteinase-activated receptor 2, c-jun N-terminal protein kinase, TNFα, Protein kinase C

## Abstract

In this study we examined the potential for PAR_2_ and TNFα to synergise at the level of MAP kinase signalling in PAR_2_ expressing NCTC2544 cells. However, to our surprise we found that activation of PAR_2_ by trypsin or the specific activating peptide SLIGKV-OH strongly inhibited both the phosphorylation and activity of JNK. In contrast neither p38 MAP kinase nor ERK activation was affected although TNFα stimulated IκBα loss was partially reversed. The inhibitory effect was not observed in parental cells nor in cells expressing PAR_4_, however inhibition was reversed by pre-incubation with the novel PAR_2_ antagonist K14585, suggesting that the effect is specific for PAR_2_ activation. SLIGKV-OH was found to be more potent in inhibiting TNFα-induced JNK activation than in stimulating JNK alone, suggesting agonist-directed signalling. The PKC activator PMA, also mimicked the inhibitory effect of SLIGKV-OH, and the effects of both agents were reversed by pre-treatment with the PKC inhibitor, GF109203X. Furthermore, incubation with the novel G_q/11_ inhibitor YM25480 also reversed PAR_2_ mediated inhibition. Activation of PAR_2_ was found to disrupt TNFR1 binding to RIP and TRADD and this was reversed by both GF109203X and YM25480. A similar mode of inhibition observed in HUVECs through PAR_2_ or P2Y2 receptors demonstrates the potential of a novel paradigm for GPCRs linked to G_q/11_, in mediating inhibition of TNFα-stimulated JNK activation. This has important implications in assessing the role of GPCRs in inflammation and other conditions.

## 1 Introduction

Proteinase-activated receptor-2 (PAR_2_), is a member of the G-protein coupled receptor (GPCR) subfamily typified by the thrombin receptor, PAR_1_ [1]. The receptor is activated by serine proteinases, most notably trypsin and tryptase [2–4]. Extensive study of PAR_2_ has revealed the receptor to be of potential importance in the functional control of blood vessel function [5,6], lung patency [7] and gastrointestinal tract motility and ion transport. A number of additional studies have implicated a role for PAR_2_ in several disease states including chronic arthritis, inflammatory pain and colitis [8–10] and in skin disorders such as type IV and contact dermatitis [11,12]. However, additional evidence also supports an anti-inflammatory role, for example PAR_2_ mediates PGI_2_-dependent relaxation of vessels which counteracts pro-inflammatory events such as ischaemia–reperfusion injury [13], and in the airways, PAR_2_ activation evokes an epithelium and prostanoid-dependent relaxation in isolated bronchi from several species [14]. What molecular mechanisms mediate such duality of function is unknown.

The cellular actions of PAR_2_ have been shown to be mediated through the activation of a number of key signalling pathways. This includes the mitogen-activated protein kinases, principally ERKs [15] mediated via interaction with the beta arrestins [16]. PAR_2_ also couples to a number of signalling pathways usually stimulated strongly by cytokines, such as the nuclear factor kappa B (NFκB) pathway and the stress-activated protein kinases, p38 map kinase and JNK [17,18]. PAR_2_ coupling to the stress-activated protein kinases, JNK, and p38 MAP kinase has been demonstrated in transfected cells [17], human blood eosinophils, and rat pancreatic stellate cells [19,20], however the exact mechanisms involved remain unclear.

Previous studies have demonstrated that activation of PAR_2_ leads to the release of several potent pro-inflammatory mediators, including IL-6, IL-8, TNFα and IL-1β [21–23], resulting in enhanced inflammatory responses. TNFα pre-treatment has been shown to up-regulate functional PAR_2_ in endothelial cells [24,25], whilst recent studies have shown PAR_2_ to interact with TLR-4 signalling via myd88 [26], suggesting the potential for PAR_2_ to enhance inflammation. Thus, in this study we examined the potential for PAR_2_ to synergize with TNFα in the activation of pro-inflammatory signalling responses such as JNK and p38 MAP kinase in a cell line (clone G) expressing both PAR_2_ and TNFR1 receptors. However to our surprise we found that activation of PAR_2_ was associated with a marked inhibition in TNFα stimulated JNK phosphorylation and to a lesser extent NFκB which was mediated by activation of the G_q/11_ pathway via the activation of PKC and had a site of inhibition at the coupling of TNFR1 to receptor associated proteins. This extends a previous study from our laboratory examining purinergic P2Y receptors and suggests the existence of a novel paradigm for GPCR signalling, the inhibition of TNFα-stimulated JNK.

## 2 Material and methods

### 2.1 Reagents

All materials used were of the highest commercial grade available and were purchased from Sigma (Dorset, UK) or Calbiochem (Nottingham, UK), unless otherwise stated. The PKC inhibitor, GF109203X, was obtained from Calbiochem (San Diego, CA, USA). Antibodies raised against phosphorylated forms of JNK and p38 MAP kinase were purchased from Biosource (CA, USA). Anti-IκBα, anti-JNK and p38 MAP kinase antibodies were obtained from Santa Cruz Biotechnology (CA, USA). Meanwhile, tumour necrosis factor-α (TNFα) was purchased from Insight Biotechnology (Middlesex, UK). The PAR_2_ activating peptide 2f-LIGKV-OH and the novel PAR_2_ peptide mimetic K-14585 were kind gifts from the Kowa Company Ltd. (Tokyo, Japan). The YM-254890 compound was a kind gift of Astellas Pharma. Inc, Japan.

### 2.2 Cell culture

Human skin epithelial cells NCTC2544 were maintained in M199 medium with Earl's salt supplement, 10% (v/v) foetal calf serum, L-glutamine (27 µg/ml), penicillin (250 U/ml), and streptomycin (25 mg/ml). NCTC2544 cells stably expressing human PAR_2_ (clone G) were maintained in complete M199 medium containing 400 μg/ml of Geneticin for selection pressure and passaged using Versene (2% EDTA/PBS). EAhy-926 cells were produced by hybridizing human umbilical vein endothelial cells (HUVECs) with the epithelial cell line A549 [27] and maintained in Dulbecco's Modified Eagle's Medium (DMEM) supplemented with 10% foetal calf serum (FCS), hypoxanthine/aminopterin/thymidine (HAT supplement), penicillin (250 U/ml) and streptomycin (25 mg/ml), and cells were passaged using Versene.

HUVECs were grown in endothelial basal media, supplemented with endothelial growth media (EGM-2) containing single aliquots (2% foetal bovine serum, 0.2 ml hydrocortisone, 2 ml hFGF-B, 0.5 ml VEGF, 0.5 ml R3-insulin like growth factor-1, 0.5 ml ascorbic acid, 0.5 ml hEGF, 0.5 ml GA 1000, and 0.5 ml heparin) purchased from Cambrex. All experiments were performed between passages 3 and 7. All cells were kept in a humidified atmosphere containing 5% CO_2_ at 37 °C.

### 2.3 Western blotting

Proteins were separated by 10% SDS–PAGE and transferred onto nitrocellulose. The membranes were blocked for non-specific binding for 2 h in 2% BSA (w/v) diluted in NATT buffer (50 mM Tris–HCl, 150 mM NaCl, 0.2% (v/v) Tween-20). The blots were then incubated overnight with 50 ng/ml primary antibody diluted in 0.2% BSA (w/v) in NATT buffer. The blots were washed with NATT buffer for 90 min and incubated with HRP-conjugated secondary antibody (20 ng/ml in 0.2% BSA (w/v) diluted in NATT buffer) for 2 h. After a further 90 min wash, the blots were subjected to ECL reagent and exposed to Kodak X-ray film.

### 2.4 Immuno-precipitation

For immuno-precipitation of TNFR1, equal amounts of pre-cleared cell lysates were incubated in Tris–HCl buffer, pH 7.4, containing 50 mM NaCl, 1 mM EDTA, 1 mM EGTA, 1% (w/v) Triton X-100, and 0.5% (w/v) Nonidet P-40, with 2 μg TNFR1 antibody, pre-coupled to protein G, for 3 h. Lysates were recovered by sequential washes in solubilisation buffer and in the same buffer lacking detergents. Precipitates were assessed for TRADD or RIP content using Western blotting.

### 2.5 JNK activity assay

To measure JNK activity, cells were stimulated as appropriate and the reaction terminated by rapid aspiration and the addition of ice-cold PBS. The cells were solubilised in 20 mM HEPES buffer, pH 7.7, containing 50 mM NaCl, 0.1 mM EDTA, 0.1 mM Na_3_VO_4_, 0.1 mM PMSF, 10 mg/ml aprotinin, 10 mg/ml leupeptin, and 1% (w/v) Triton X-100. Lysates were clarified by centrifugation for 5 min at 13,000 rpm and equal amounts of protein were incubated with 20 μg of GST-c-Jun-(5–89) immobilized on glutathione-Sepharose at 4 °C for 3 h. Beads were then washed three times in solubilisation buffer and twice in 25 mM HEPES buffer, pH 7.6, containing 20 mM β-glycerophosphate, 0.1 mM NaV_3_O_4_, and 2 mM dithiothreitol. Precipitates were then incubated with the same buffer containing 25 μM/0.5 μCi of ATP/[γ-^32^P] ATP in a final volume of 30 µl at 30 °C for 30 min. The reactions were terminated by the addition of 4× SDS-sample buffer and aliquots of each sample subjected to electrophoresis on 11% SDS-polyacrylamide gel electrophoresis. Phosphorylation of GST-c-Jun was then determined by autoradiography.

### 2.6 Statistical analysis

Where experimental data is shown as a blot, this represents one of at least 3 experiments and data represents the mean ± s.e.m. Statistical analysis was by one-way ANOVA with Dunnett's post test (*P < 0.05, **P < 0.01).

## 3 Results

**Fig. 1:**
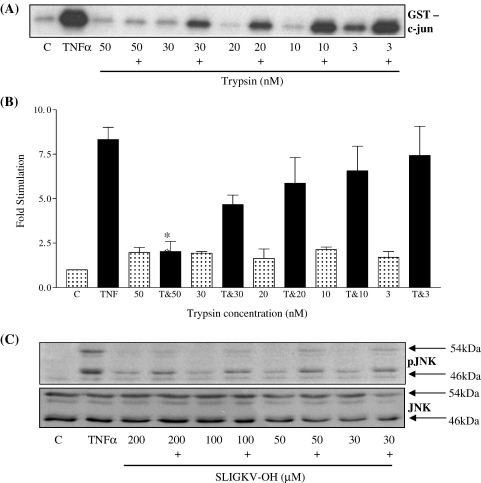
PAR_2_ activation mediates inhibition of TNFα-stimulated JNK activity and phosphorylation in PAR_2_ NCTC2544 cells. PAR_2_ expressing NCTC2544 cells (clone G) were pre-incubated with increasing concentrations of trypsin or SLIGKV-OH for 30 min prior to stimulation for a further 30 min with TNFα (+) (10 ng/ml). Samples were assessed for JNK activity (Panel A) or phospho-JNK levels (Panel C) as outlined in the Methods section. Gels from JNK assays were quantified (Panel B). Each value represents the mean ± s.e.m from at least 4 experiments and data was quantified by densitometry. ⁎*P* < 0.05, ⁎⁎*P* < 0.01 compared to TNFα alone.

Initially we sought to investigate the potential for synergy between PAR_2_ and TNFα receptor subtypes by a combination of different pre-incubation strategies. However no noticeable synergy was observed; rather when cells were pre-incubated with trypsin for 30 min we revealed a concentration-dependent inhibition of subsequent TNFα-stimulated JNK activity, as measured by *in vitro* c-Jun phosphorylation (Fig. 1, Panels A and B). This inhibitory effect was mimicked by the human PAR_2_ tethered ligand agonist SLIGKV-OH and was reflected at the level of phospho-JNK confirming inhibition of JNK phosphorylation rather than JNK activity itself (Panel C).

**Fig. 2:**
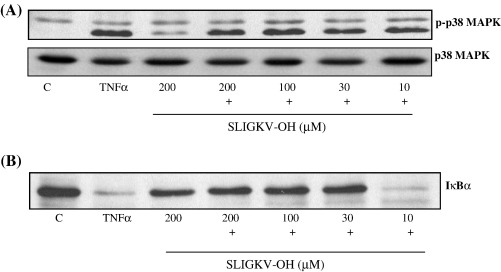
The effect of SLIGKV-OH upon TNFα-stimulated p38 MAP kinase phosphorylation and IκBα loss. PAR_2_ expressing NCTC2544 cells (clone G) were pre-incubated with increasing concentrations of SLIGKV-OH for 30 min prior to stimulation for a further 30 min with TNFα (+) (10 ng/ml). Samples were assessed for phospho-p38 MAP kinase activity (Panel A) or IκBα levels (Panel B) as outlined in the Methods section. Each gel is representative of at least 4 experiments.

Inhibition of TNFα signalling was pathway specific (Fig. 2), as no equivalent inhibition of p38 MAP kinase was observed following SLIGKV-OH pre-treatment (Panel A) whilst ERK activation in response to TNFα was negligible in this cell type (data not shown). However, TNFα-induced loss in IκBα expression, a marker of NFκB activation, was partially reversed (Fig. 2, Panel B).

**Fig. 3:**
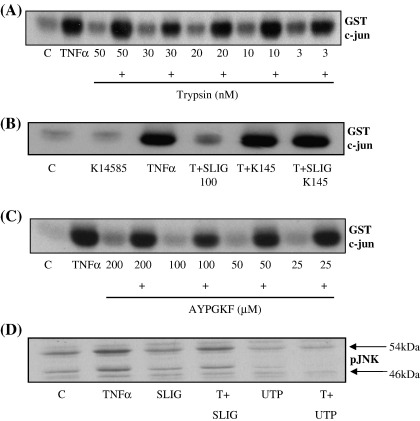
Inhibition of TNFα-stimulated JNK activity is dependent upon PAR_2_ activation. In Panel A, parental NCTC2544 cells were pre-incubated with increasing concentrations of trypsin, 30 min prior to stimulation for a further 30 min with TNFα (+) (10 ng/ml). In Panel B, PAR_2_ NCTC2544 cells were pre-incubated with 10 μM K14585 for 30 min prior to addition of SLIGKV-OH (100 μM) for 30 min and stimulation with TNFα. In Panel C, PAR_4_ expressing cells were incubated with AYPGKF (100 µM) 30 min prior to TNFα stimulation. In Panel D, HUVECs were treated with either SLIGKV-OH (SLIG) or 50 μM UTP for 30 min prior to TNFα stimulation. Samples were assessed for JNK activity or phospho-JNK content as outlined in the Methods section. Each gel is representative of at least 3 experiments.

We next sought to confirm that PAR_2_ was indeed required for tryspin and peptide mediated inhibition of TNFα-mediated JNK signalling (Fig. 3). Using either parental NCTC2544 or vector expressing cells (not shown) we found no equivalent inhibition of JNK activity (Panel A) at any concentration of peptide tested. Secondly, we found that pre-incubation of cells with the novel PAR_2_ antagonist K-14585 was able to reverse the inhibition of JNK mediated by the human PAR_2_ activating peptide SLIGKV-OH (Panel B). Another candidate PAR, PAR_4_ expressed in the same cell type was without effect confirming the receptor specificity of the response (Panel C). Panel D illustrates that this inhibition is a feature of endogenously expressed PAR_2_, as pre-treatment of HUVECs with peptide reduced TNFα-stimulated JNK phosphorylation. Interestingly, P2Y2 stimulation mediated greater inhibition of TNFα stimulated JNK signalling, suggesting that other GPCRs exhibit the same phenomenon.

**Fig. 4:**
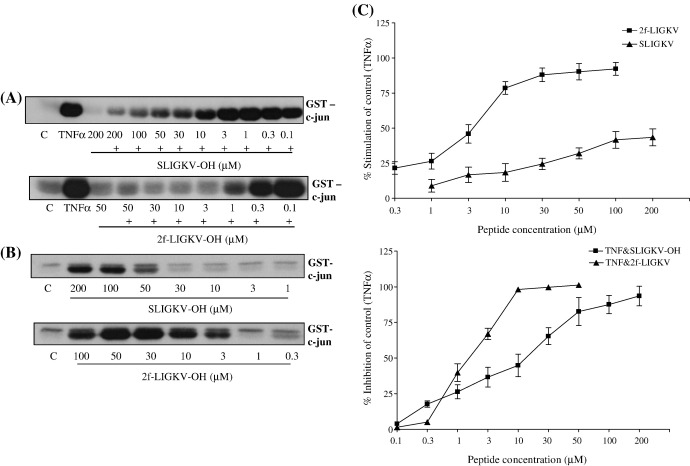
Concentration response effects of PAR_2_ peptides for activation of JNK and inhibition of TNFα-stimulated JNK. PAR_2_ expressing NCTC2544 cells (clone G) were incubated with increasing concentrations of SLIGKV-OH or 2f-LIGKV-OH for 30 min and assayed for JNK activity (Panel B) or stimulated for a further 30 min with TNFα (+) before JNK activity was assessed (Panel A). Samples were assessed for JNK activity as outlined in the Methods section. Gels from JNK assays were quantified (Panel C). Each value represents the mean ± s.e.m from at least 4 experiments and data was quantified by densitometry.

In addition, we further analysed the concentration dependency of not only SLIGKV-OH but also the substituted peptide 2f-LIGKV-OH originally identified by ourselves as a more potent PAR_2_ agonist [8,28]. Both peptides profoundly inhibited TNFα-stimulated JNK activity in a concentration-dependent manner with IC_50_ values of 6 μM (6.24 ± 1.319 μM) for SLIGKV-OH and approximately 2 μM (1.582 ± 0.3422 μM) for 2f-LIGKV-OH (Fig. 4, Panel A). This accords well with the potency of these peptides assessed in a series of assays using the same cell line [28]. However, we also found that these peptides alone were able to stimulate JNK activity in a concentration-dependent manner (Panel B). For example, 2f-LIGKV-OH caused a strong activation of JNK with an EC_50_ value of 2 μM (2.121 ± 0.4041 μM), similar to its ability to inhibit JNK stimulation in response to TNFα. In contrast, SLIGKV-OH was a less potent activator of JNK with an EC_50_ value of approximately 10 μM (10.12 ± 3.305 μM) in comparison to its ability to inhibit JNK (Panel C). Moreover, the difference in efficacy was marked, and SLIGKV-OH behaved as a full agonist in inhibiting TNFα signalling (% inhibition = 93.567 ± 6.882%) but was a partial agonist in stimulating JNK activity alone (% stimulation = 43.950 ± 6.482%).

**Fig. 5:**
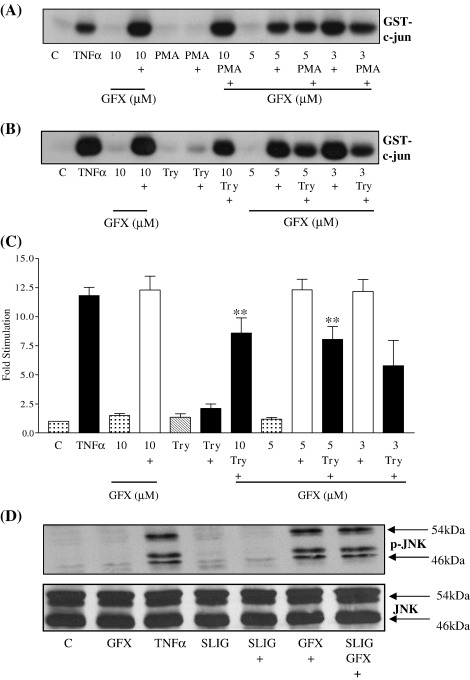
Pharmacological blockade of protein kinase C reverses PAR_2_ mediated inhibition of TNFα-stimulated JNK activation. PAR_2_ expressing NCTC2544 cells (clone G) were pre-incubated with increasing concentrations of GF109203X (GFX) for 30 min then incubated for a further 30 min with 100 nM PMA (Panel A) or 30 nM trypsin (Panel B) prior to stimulation for a further 30 min with TNFα(+) (10 ng/ml). Samples were assessed for JNK activity (Panels A and B) or phospho-JNK levels (Panel D) as outlined in the Methods section. Gels from JNK activity assays were quantified by densitometry (Panel C). Each value represents the mean ± s.e.m from at least 4 experiments. ⁎⁎*P* < 0.01 compared to TNFα and trypsin stimulated.

We then examined the potential signalling pathways which could be mediating the inhibitory effects of PAR_2_. PAR_2_ is coupled to a number of signalling pathways via G-protein dependent and independent mechanisms. Initially, we found that we could mimic the inhibitory effect of PAR_2_ activation with PMA and that this effect could be reversed by micromolar concentrations of the PKC inhibitor, GF109203X, thereby implicating the involvement of PKC isoforms (Fig. 5, Panel A). Therefore, we tested the effect of the PKC inhibitor GF109203X on PAR_2_ mediated inhibition of JNK activity and phosphorylation. Pre-incubation with a maximal concentration (10 μM) of this inhibitor reversed the inhibitory effect of trypsin and SLIGKV-OH on TNFα-stimulated JNK activation (Panel B and C) and phosphorylation (Panel D). In contrast, another inhibitor Go6976 which preferentially inhibits the classical group of PKC isoforms was without effect (not shown) suggesting a potential role for the novel PKC isoforms.

**Fig. 6:**
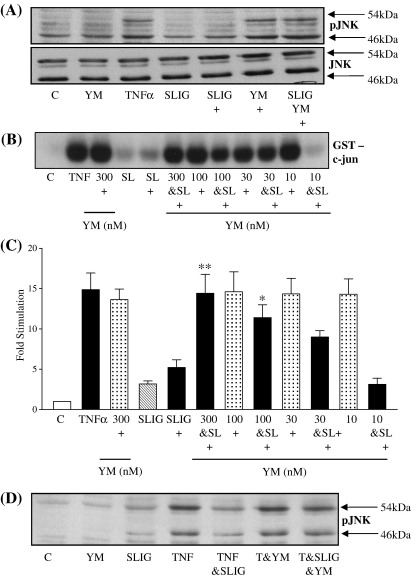
G_q/11_ is essential for PAR_2_ mediated inhibition of JNK activation by TNFα. PAR_2_ expressing NCTC2544 cells (Panels A and B) or HUVECs (Panel D) were pre-incubated with (100 nM — Panel A) increasing concentrations of YM254890 (YM) for 30 min then incubated for a further 30 min with SLIGKV-OH (100 μM) prior to stimulation for a further 30 min with TNFα (+) (10 ng/ml). Samples were assessed for phospho-JNK levels (Panels A and D) or JNK activity (Panel B) as outlined in the Methods section. Gels from JNK activity assays were quantified by densitometry (Panel C). Each value represents the mean ± s.e.m from at least 4 experiments. ⁎⁎*P* < 0.01 compared to TNFα and peptide stimulated.

We then examined the role of Gα_q/11_ in the PAR_2_ mediated inhibitory effect (Fig. 6), using the novel Gα_q/11_ inhibitor YM254890. This compound has been used previously in our laboratory and has been validated elsewhere [29]. In untreated cells, TNFα stimulated JNK phosphorylation and activity as expected, and this was inhibited by the pre-treatment with SLIGKV-OH. However pre-treatment of the cells with YM254890 resulted in almost complete abrogation of peptide mediated inhibition of TNFα stimulated JNK phosphorylation (Panel A) and activity (Panels B and C). A similar reversal was also observed in HUVECs (panel D) although the initial PAR_2_ mediated inhibition was less.

**Fig. 7:**
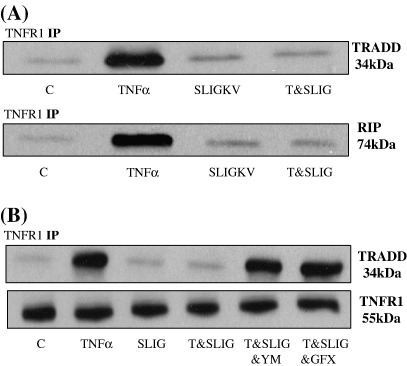
PAR_2_ mediated TNFR1 adapter protein disruption in PAR_2_ expressing cells. In Panel A, cells were pre-treated with peptide (100 μM) or vehicle prior to stimulation with TNFα (10 ng/ml) for 30 min. Lysates were immuno-precipitated for TNFR1 and immuno-blotted for TRADD and/or RIP, as outlined in the Methods section. In Panel B cells were pre-treated with 100 nM YM254890 or 3 μM GF109203X for 30 min prior to pre-treatment with peptide (100 μM), for 30 min, then incubated for a further 30 min with TNFα (10 ng/ml). TNFR1 immuno-precipitates were immuno-blotted for TRADD. Each gel is representative of at least three experiments.

We next examined the potential site of inhibition within the TNFα-mediated cascade. We assessed the potential for PAR_2_ activation to disrupt the interaction of TNFR1 with a number of associated proteins. TNFR1 was precipitated and the interaction with other proteins assessed by Western blotting. In basal conditions binding to TRADD and RIP was minimal, however, following stimulation with TNFα there was a marked increase in both TRADD and RIP recovery confirming the formation of a TNFα-sensitive complex. Formation of this complex was severely disrupted with PAR_2_ peptide pre-treatment (Fig 7, Panel A). Using the TNFR1/TRADD interaction as a model we pre-incubated cells with either GF109203X or YM254890 prior to PAR_2_ activating peptide pre-treatment. Both agents effectively reversed PAR_2_ mediated disruption of TNFα driven TNFR1 and TRADD complex formation (Panel B).

## 4 Discussion

This study identifies a novel paradigm for PAR_2_ inhibition of TNFα-induced JNK signalling. Our evidence strongly points to a G_q/11_ and PKC-dependent cascade which functions to disrupt TNFR1 dependent signalling. Significantly, this inhibitory phenomenon is a feature of PAR_2_ in cells endogenously expressing the receptor and in response to activation of other GPCRs, namely P2Y2 receptors. Thus, it may have implications as to the regulatory role of GPCRs in a number of physiological and pathophysiological conditions.

Initially, we sought to assess the potential of synergy between PAR_2_ activation and other cytokines known to participate in inflammation such as TNFα, IL-1 and LPS through activation of selective TLRs. Indeed a recent study has demonstrated synergy between PAR_2_ and TLR-4 through the activation of Myd88 [26], and a synergistic effect between the PAR-2 and LPS receptor, was also previously demonstrated where PAR agonists and LPS in combination potentiated the synthesis of IL-8 from airway epithelial cells [30]. However while research to date has shown the possibility of receptor cross talk between the PAR family members [31] and with other GPCRs such as the epidermal growth factor receptor (EGFR) [32] few studies have looked at possible interactions with other receptors, such as cytokine, chemokine and other inflammatory receptors. Since activation of PAR_2_ exhibits many features similar to cytokines regarding the activation of inflammatory mediator release, this study sought to characterise the differences and similarities of the signalling pathways mediated by both PAR_2_ and TNFα, and to examine the possibility of synergy between these two receptors. To our surprise we found that PAR_2_ activation was able to strongly mediate an inhibition of TNFα mediated JNK signalling in response to cellular activation by trypsin suggesting a PAR_2_ mediated response. Indeed the inhibitory effect of trypsin was mimicked with the peptides SLIGKV-OH and 2f-LIGKV-OH, but reversed by a novel PAR_2_ antagonist K14858, which we have recently characterised [33].

Our pharmacological studies also revealed the potential for agonist-mediated signalling in relation to PAR_2_ and JNK. We have previously demonstrated that trypsin mediated activation of PAR_2_ results in an increase in JNK activity [17] and indeed alone SLIGKV-OH and 2fl-LIGKV-OH were able to increase JNK. However, we also found a distinct profile in the ability of SLIGKV-OH to inhibit TNFα mediated JNK and to activate JNK alone, it was less potent and efficacious in the latter assay. Thus, we can discern two agonist-mediated directed events which relate to the activation of JNK. This type of modality is emerging for GPCRs for example the β2 antagonist propranolol, whilst being an inverse agonist at the level of cAMP accumulation, functions as a partial agonist at the level of ERK activation [34]. Similar types of dual coupling to ERK and other intermediates have recently been demonstrated for PAR_2_ [35,36] but not due to agonist specific differences.

Whilst this present study focuses on two agonist effects it is the first study to raise this potential when examining JNK activation. This model was also supported when examining the effect of the novel PAR_2_ antagonist K-14585 [33]. Whilst in this present study we show that K-14585 is able to reverse PAR_2_ mediated inhibition of TNFα signalling, we found that at higher concentrations, K14585 alone is able to activate both JNK (not shown) and p38 MAP kinase [37]. Taken together, these findings indicate two distinct agonist-directed signalling pathways driven by SLIGKV-OH, one dependent upon G_q/11_ and one independent, mediating inhibition and activation of JNK respectively. Studies assessing JNK activation in response to GPCRs are limited; however, it has been shown that free βγ dimers and Gα_12/13_ subunits can activate JNK in a Rac1-cdc42 dependent manner [38]. Gα_12_ can also activate JNK via ASK1, independently of the activation of Rac1 and cdc42 [39].

We then sought to investigate the possible mechanisms by which PAR_2_ mediated inhibition of JNK might occur. Our initial studies indicated that DAG-dependent PKC isoforms are involved in the response since PMA was able to mimic the effect of PAR_2_ activation, abolishing both JNK activity and phosphorylation. We found good reversal of PAR_2_ mediated inhibition using GF109203X, an inhibitor known to block both Ca^2+^-dependent and some novel PKC isoforms [40–42]. NCTC2544-PAR_2_ cells were found to express the PKC isoforms, PKCα, β11, δ, ε and θ. This is typical of a number of keratinocyte cell types including NHEK and HaCat [43]. Previously, we have shown that PKCα and PKCδ play a role in regulating NFκB signalling at the level of both IKK activation and phosphorylation of p65 NFκB, however equivalent studies here using siRNA for PKCα, ε and θ have not revealed a specific isoform to date responsible for JNK inhibition (not shown). PKC is also implicated in a number of other PAR_2_-mediated events including sensitization of the TRPV1 in rats and mice, and chloride transport in intestinal epithelial cells [44,45] and this current study extends the role for PKC in PAR_2_-dependent signalling events.

Our studies also revealed an important role for G_q/11_ in PAR_2_ mediated inhibition of JNK signalling. We used the novel G_q/11_ inhibitory compound YM254890 which we have previously shown to be a very effective inhibitor of trypsin and peptide mediated [^3^H]IP accumulation [29]. In the present study we demonstrated complete reversal of PAR_2_ mediated JNK inhibition. Thus, DAG derived from G_q/11_ activation is likely to drive PKC activation and subsequent inhibition of TNFα stimulated JNK activity. Previously, we have shown that G_q/11_ plays a positive role in the regulation of PAR_2_ mediated NFκB phosphorylation and in DNA reporter activity [29], whilst other groups have clearly demonstrated PAR_2_ coupling to NFκB. Thus, these findings conflict with this present study showing the potential of G_q/11_ to play a role in PAR_2_ mediated inhibition of TNFα stimulated IκBα loss. Interestingly, whilst we were able to demonstrate PAR_2_ mediated inhibition of TNFα induced IκBα loss this effect was not manifest further downstream, and pre-treatment of cells with peptide did not inhibit subsequent TNFα-stimulated NFκB reporter activity. Thus, because of the strong interactions of PAR_2_ with NFκB [29], the overall outcome of PAR_2_/TNFR1 is a loss in JNK activation only. We are currently assessing if receptors more poorly coupled to NFκB, for example P2Y2, can mediate an overall inhibition of NFκB activation.

We also demonstrate for the first time receptor mediated driven disruption of TNFR1 binding to a number of associated proteins. Upon activation TNFR1 associates with a number of proteins including TRADD, RIP, TRAF2, MADD and FADD [46]. Formation of this complex at the membrane allows TNFα to mediate an array of different signalling events, such as induction of apoptosis through the FADD-caspase arm or activation of MAP kinase and NFκB signalling via TRAF2. For example, TRAF2 contains a conserved C-terminal region termed the TRAF domain, which interacts with TRADD and an N-terminal ring finger required for signalling the activation of NFκB and JNK/SAPK [47–49]. The fact that p38 MAP kinase was not inhibited suggests a point in the pathway upstream of both NFkB and JNK. This is likely to be at the level of TRAFs or MEKKs such as TAK1 or ASK1, however this will be difficult to decipher as less is known about TRAF mediated activation of the p38 MAPK pathway [46]. Two recent studies have shown the potential of PMA, a PKC activator, to disrupt TNFR1 binding to these intermediates [42,50], however the study presented here is the first to show that this can be receptor mediated. Pharmacological experiments also demonstrate that the PAR_2_/G_q/11_/PKC axis is essential in driving such disruption in response to PAR_2_. At this point the exact mechanism mediating disruption is unclear, no studies to date have identified any PKC phosphorylation sites within the TNFR1 complex, and thus it is possible that additional intermediate proteins may be involved.

The potential for GPCRs to mediate the inhibition of TNFα-mediated JNK signalling has clear physiological significance. For example, in the endothelium, activation of PAR_2_ would be able to reduce the damaging effects of TNFα during inflammation. In other cell types where PAR_2_ is expressed inhibition of TNFR1 signalling may be of significance in relation to cancer.

## 5 Conclusions

In conclusion we have demonstrated for the first time a novel mechanism whereby PAR_2_, a GPCR can disrupt TNFα mediated activation of the JNK pathway. This will provide valuable information regarding the role of GPCRs in the context of inflammation and other disease states.

Specific points:

1. PAR_2_ mediated inhibition of TNFα activated JNK is via coupling to the heterotrimeric G-protein, G_q/11_.
2. This inhibitory response requires members of the PKC family, and inhibits TNF activation of JNK by disrupting coupling of accessory proteins, such as RIP and TRADD to TNFR1.
3. This inhibitory effect is mediated by other Gα_q/11_ linked GPCRs, in particular by the P2Y2 receptor.
